# Molecular Characterization of Rift Valley Fever Virus From the 2025 Outbreak in Northern Senegal Reveals Lineage H Persistence and Key Polymerase Mutations

**DOI:** 10.1002/jmv.70734

**Published:** 2025-11-30

**Authors:** Moussa Moïse Diagne, Gamou Fall, Abiboulaye Sall, Bocar Sow, Ndeye Awa Ndiaye, Alioune Gaye, Mamadou Sarr Ndao, Aboubacry Gaye, El Hadji Ndiaye, Mignane Ndiaye, Seynabou Mbaye Ba Souna Diop, Safiétou Sankhe, Mouhamed Kane, Seynabou Ndiaye, Ousmane Faye, Yoro Sall, Mamadou Aliou Barry, Ibou Gueye, Marie Henriette Dior Ndione, Boly Diop, Ousmane Cisse, Joseph R. A. Fitchett, Ibrahima Dia, Cheikh Loucoubar, Ndongo Dia, Mawlouth Diallo, Boubacar Diallo, Ibrahima Soce Fall, Mamadou Ndiaye, Diawo Diallo, Abdourahmane Sow, Oumar Faye

**Affiliations:** ^1^ Virology Department Institut Pasteur de Dakar Dakar Senegal; ^2^ Ministry of Health and Public Hygiene Dakar Senegal; ^3^ Public Health Direction Institut Pasteur de Dakar Dakar Senegal; ^4^ Department of Public Health and Preventive Medicine, Faculty of Medicine, Pharmacy and Odonto‐stomatology Université Cheikh Anta Diop de Dakar Dakar Senegal; ^5^ Zoology Medical Department Institut Pasteur de Dakar Dakar Senegal; ^6^ Epidemiology, Clinical Research and Data Science Department Institut Pasteur de Dakar Dakar Senegal; ^7^ Biotechnology Advisory Office Institut Pasteur de Dakar Dakar Senegal; ^8^ Chief Executive Office Institut Pasteur de Dakar Dakar Senegal

**Keywords:** genomic surveillance, lineage H, Rift Valley fever virus, Senegal 2025 outbreak, viral evolution

## Abstract

Rift Valley fever virus (RVFV) is a mosquito‐borne phlebovirus that causes severe febrile and hemorrhagic illness in humans. In September 2025, an outbreak in northern Senegal led to 119 confirmed infections and 15 deaths as of October 7, 2025. We performed rapid genomic sequencing to characterize the virus responsible for this epidemic. RNA from RT‐qPCR–confirmed samples was sequenced using the Twist Comprehensive Viral Research Panel on an Illumina iSeq 100 platform. Consensus genomes were analyzed with and compared with all complete RVFV genomes in GenBank. Nine genomes were recovered, including five complete tripartite sequences. All clustered within lineage H, sharing > 99% nucleotide identity with Senegalese isolates from 2020 to 2022. Alongside two conservative mutations (R137K and K1111R in S and M segments, respectively), a single nonconservative D11N substitution in the L polymerase may affect replication efficiency, while Gn and Gc epitopes remained conserved. Phylogenetic analyses confirmed strong genetic continuity with earlier West African isolates, indicating local persistence rather than reintroduction. Lineage H persistence in Senegal, combined with polymerase substitutions under purifying selection, suggests subtle viral adaptation that may affect replication. Conserved glycoprotein epitopes indicate maintained vaccine relevance. Sustained genomic surveillance integrated with clinical and ecological monitoring remains essential to anticipate viral evolution and guide Rift Valley fever control.

## 1 Introduction and Outbreak Context

Rift Valley fever virus (RVFV), a mosquito‐borne phlebovirus of the family Phenuiviridae, was first described in Kenya in 1931 [1]. Since then, the virus has caused repeated epidemics in Africa and the Arabian Peninsula, including major outbreaks in Egypt (1977), East Africa (1997–1998, 2006–2007), Saudi Arabia, and Yemen (2000) [2]. In West Africa, recurrent epidemics have been reported in Mauritania, Niger, Mali, and Senegal [3, 4].

Senegal has a well‐documented history of RVF outbreaks. The first major outbreak occurred in 1987, at the frontier of Senegal and Mauritania, producing ~1500 human cases with 200 deaths [5]. Additional outbreaks or sporadic cases were reported in 1993–1994, 1998–1999, 2002–2003, 2012, and 2013–2014 but the impact was very minor without death [6]. In recent years, genomic studies have shown that lineage H, previously detected in Southern Africa, became established in Senegal from 2020 onward, replacing older West African lineages [7, 8].

In September 2025, an outbreak of RVF was confirmed in the Saint‐Louis region of northern Senegal. The first confirmed cases were identified on September 20 at the regional hospital. By September 26, 11 cases, including 4 deaths, had been confirmed. As investigations expanded, the outbreak spread to Matam and Louga regions. In parallel, veterinary surveillance reported an increase in abortions among small ruminants in the Saint‐Louis area, consistent with active RVFV circulation in livestock.

The Ministry of Health classified the event as a severe epidemic and activated the Regional Epidemic Management Committee with an Incident Management System to coordinate response. By October 7, surveillance data indicated more than 600 suspected cases, of which 100 were confirmed by RT‐qPCR and 19 by IgM serology, giving 119 laboratory‐confirmed infections and 15 deaths overall.

Given the severe clinical presentation, the Institut Pasteur de Dakar conducted rapid genomic sequencing of patient samples to assess whether viral genetic changes could explain the outbreak's lethality.

## 2 Materials and Methods

RNA from RT‐qPCR–confirmed samples was sequenced on an Illumina iSeq 100 using the Twist Comprehensive Viral Research Panel hybrid‐capture workflow, following the manufacturer's instructions as previously described [8].

For genome assembly, raw FASTQ reads were trimmed with Trimmomatic (v0.39) and L, M, and S segments aligned to references using BWA‐MEM (v0.7.17). Consensus sequences were generated with iVar (v1.3.1) using a 50% allele frequency and ≥ 10× depth threshold. BAM files were annotated with LoFreq indelqual, and SNVs/indels called using LoFreq (v2.1.5). Genomes were considered complete when more than 90% of the reference segment length was recovered at a minimum mean depth of 10× across each segment.

Phylogenetic analyses were performed by combining 2025 outbreak genomes with all complete and near‐complete RVFV genomes available in GenBank. Alignments were produced in MAFFT, and maximum‐likelihood trees reconstructed using IQ‐TREE with ModelFinder‐selected substitution models and 1000 ultrafast bootstrap/SH‐aLRT replicates. Trees were visualized in iTOL [9]. Mutations were examined in AliView [10], distinguishing synonymous from nonsynonymous changes via codon display and confirmed with MEGA (Nei–Gojobori method, codon‐based *Z*‐test of selection [11]).

## 3 Results and Discussion

Nine RVFV genomes spanning the L, M, and S segments were assembled. Coverage exceeded 90% for five complete genomes (S1, S2, S3, S4, S8), while others showed partial coverage (Table 1). For analysis, only sequences with ≥ 15× mean depth were retained, yielding five complete genomes and one additional M segment (S5).

**Table 1:** Sequencing coverage of RVFV genomes from the 2025 outbreak in Senegal.

ID‐sequencing	ID‐diagnostic	RVFV segment	Coverage (%)	Depth (×)
S1	471872	L*	99.86	1813.38
		M*	99.85	2645.57
		S*	99.76	4959.01
S2	473088	L*	99.86	2416.7
		M*	99.82	4182.69
		S*	99.7	4231.65
S3	471871	L*	99.81	64.786
		M*	99.41	91.3745
		S*	99.41	81.198
S4	473086	L*	99.81	850.33
		M*	99.67	902.365
		S*	98.93	2006.94
S5	473084	L	69.94	9.18785
		M*	95.73	12.2494
		S*	94.38	9.2145
S6	473113	L	0.0	0.0
		M	0.0	0.0388674
		S	0.0	0.0
S7	471869	L	31.48	4.11438
		M	60.95	6.71995
		S	44.79	5.27395
S8	473112	L*	99.81	100.237
		M*	99.43	173.675
		S*	99.41	131.082
S9	471625	L	16.94	2.84971
		M	46.0	5.1861
		S	57.51	7.4911
S10	471624	L	51.76	7.1008
		M*	94.95	13.1504
		S	78.05	8.09735

*Note:* * Indicates genome with at least 90% horizontal coverage.

**Figure 1:**
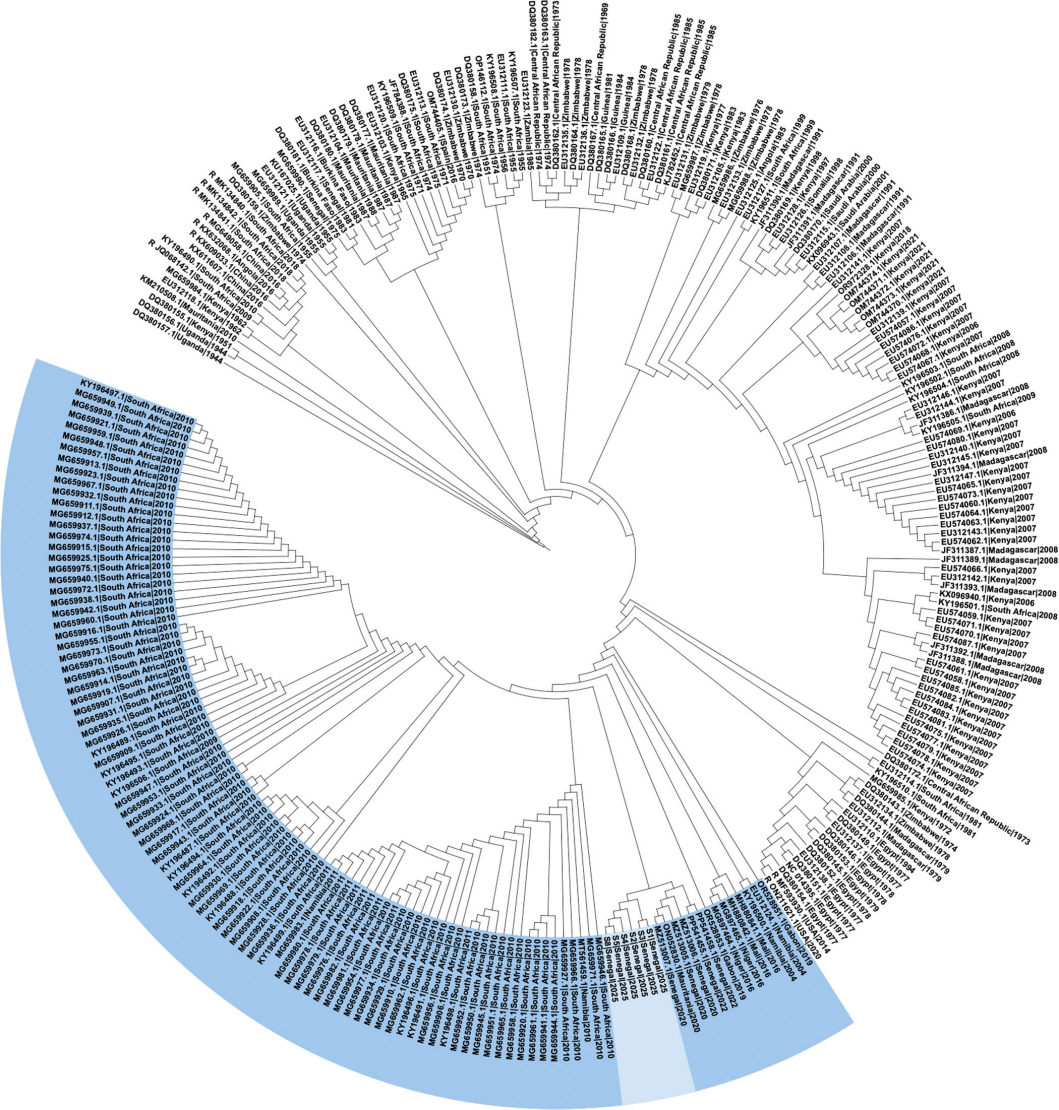
Maximum‐likelihood phylogenetic trees of the S segment constructed from all complete and nearly complete Rift Valley fever virus genomes available in GenBank. Sequences belonging to lineage H (highlighted) include the 2025 Senegal outbreak genomes (lighter shade), which cluster closely with isolates from Fatick (2020), Matam (2022), and Mauritania (2020), supporting continued local circulation of this lineage rather than a novel introduction.

**Figure 2:**
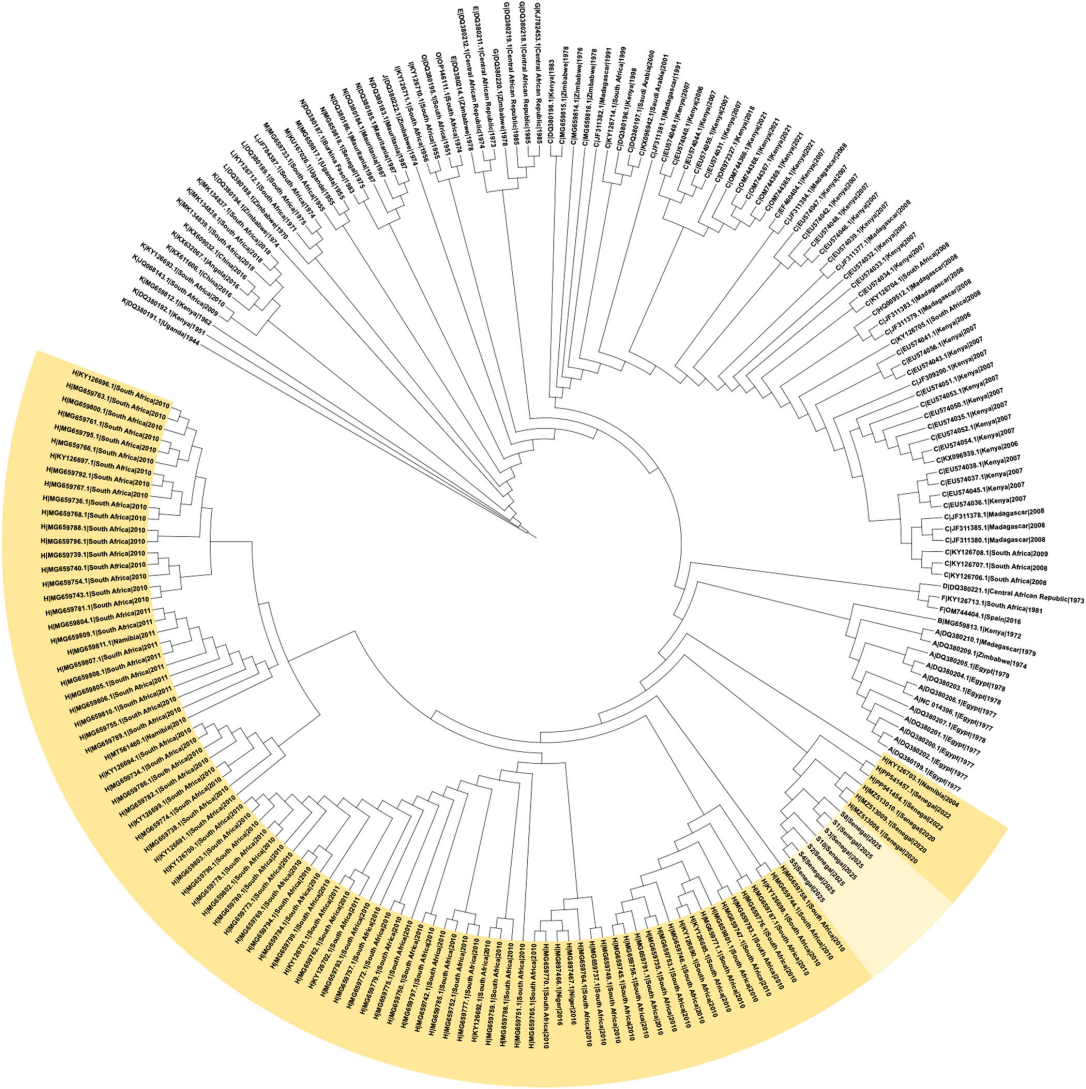
Maximum‐likelihood phylogenetic trees of the M segment constructed from all complete and nearly complete Rift Valley fever virus genomes available in GenBank. Sequences belonging to lineage H (highlighted) include the 2025 Senegal outbreak genomes (lighter shade), which cluster closely with isolates from Fatick (2020), Matam (2022), and Mauritania (2020), supporting continued local circulation of this lineage rather than a novel introduction.

**Figure 3:**
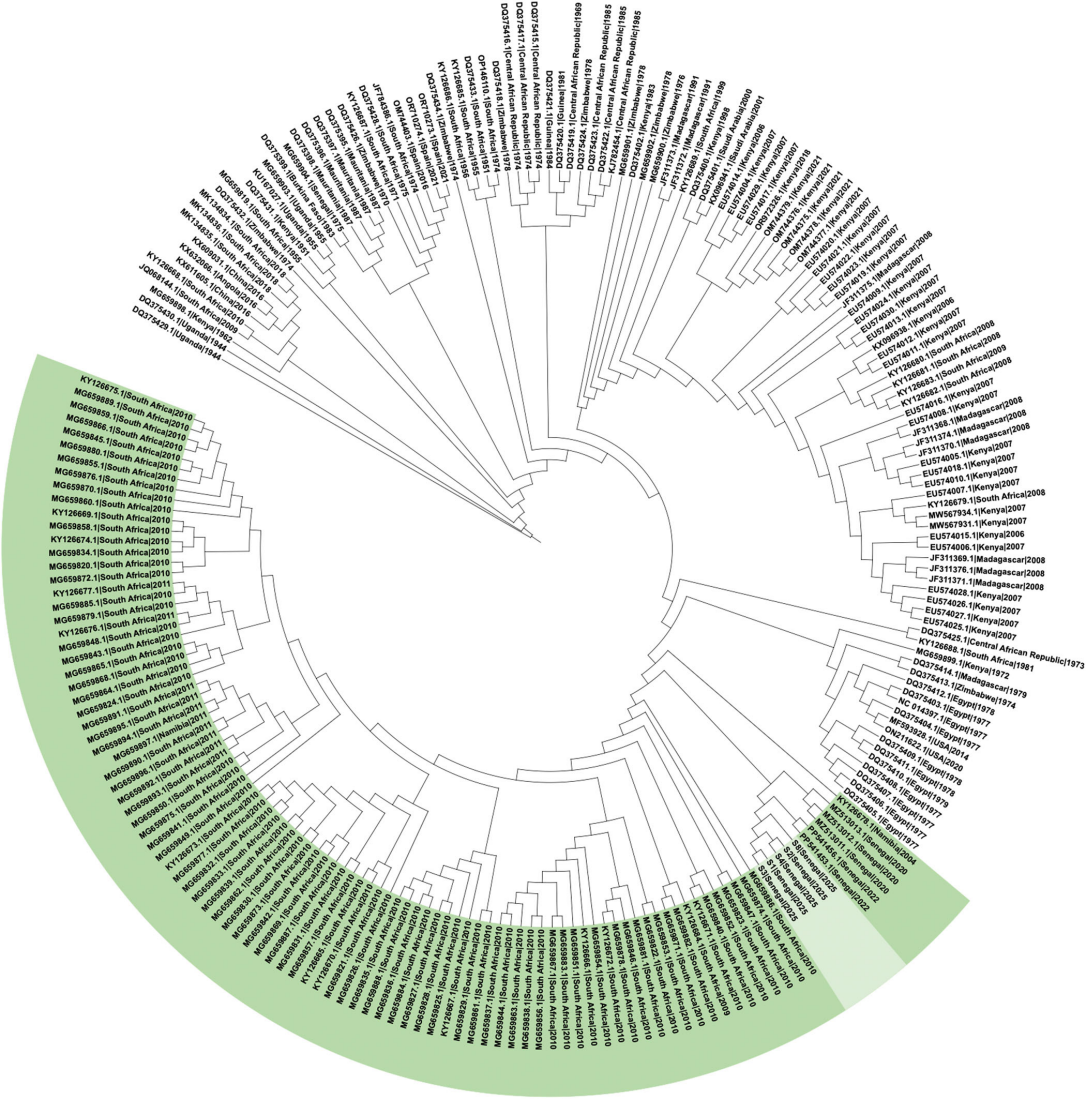
Maximum‐likelihood phylogenetic trees of the L segment constructed from all complete and nearly complete Rift Valley fever virus genomes available in GenBank. Sequences belonging to lineage H (highlighted) include the 2025 Senegal outbreak genomes (lighter shade), which cluster closely with isolates from Fatick (2020), Matam (2022), and Mauritania (2020), supporting continued local circulation of this lineage rather than a novel introduction.

Comparative analysis showed that the 2025 outbreak genomes shared more than 99% nucleotide identity with Senegalese isolates from Fatick (2020) and Matam (2022) [7, 8]. Phylogenetic reconstruction confirmed that these strains cluster within the West African branch of lineage H, indicating continued local circulation rather than a novel introduction (Figures 1, 2, 3, for S, M, and L segments, respectively).

Selection pressure analysis showed different evolutionary patterns across the genomes. When compared with the reference strain ZH‐548 M12, the outbreak sequences showed clear signs of positive selection (*Z *≈ –3.4 to –4; *p* < 0.01), meaning they accumulated more amino acid changes than expected. In contrast, evidence of purifying selection (*Z* > 0) was detected among sequences obtained during the 2025 outbreak, suggesting strong functional constraint and limited diversification within this epidemic cluster. Looking at changes over time, comparisons between 2020, 2022, and 2025 genomes suggested moderate adaptive trends, with slightly negative Z values (–1.5 to –1.8) but nonsignificant *p* values (0.07–0.13). Full results are provided in Tables [Link], [Link], [Link].

Most of the newly identified amino acid substitutions were conservative, including R137K in NSs and K1111R in NSm. Several changes in the L polymerase were shared with strains circulating in Senegal during 2020 and 2022 (Table 2). These substitutions involve residues of similar biochemical properties and are unlikely to significantly alter protein function [12].

**Table 2:** Amino acid mutations identified in RVFV lineage H strains from the 2025 Senegal outbreak.

Gene/segment	Mutation (AA change)	Type (conservative vs. nonconservative)	Likely effect on virus	Conclusion
NSs (S segment)	R137K (Arg → Lys)	Conservative (both basic)	Minimal effect on interferon antagonism; still functional	Marker mutation, not major functional impact
NSm (M segment)	K1111R (Lys → Arg)	Conservative (both basic)	Antiapoptotic function likely preserved	Marker mutation, little phenotypic change
L polymerase	D11N (Asp → Asn)	Nonconservative (charge lost)	Possible subtle change in N‐terminal endonuclease (cap‐snatching)	May fine‐tune replication efficiency
L polymerase	M120T^a^ (Met → Thr)	Nonconservative (hydrophobic → polar)	Could alter local folding near endonuclease	Potential minor replication effect
L polymerase	R158K^a^, R493K^a^, R1926K^a^ (Arg → Lys)	Conservative	Likely neutral for polymerase activity	Neutral substitutions
L polymerase	E177D^a^ (Glu → Asp)	Conservative	No significant effect (similar negative charge)	Neutral substitution
L polymerase	V1760I^a^ (Val → Ile)	Conservative	No major impact on hydrophobic core	Neutral substitution
Lineage H (general)	Shared synonymous mutations	Neutral	Useful for phylogenetic tracking	Serve as lineage markers

^a^ Only shared with strains circulating in Senegal in 2020 and 2022 [7, 8].

A single nonconservative D11N mutation in the L segment, unique to the 2025 outbreak strains and located in the endonuclease domain, may subtly affect cap‐snatching activity or fine‐tune replication efficiency. The M120T substitution introduces a polar residue in place of a hydrophobic methionine in the N‐terminal region, potentially influencing local folding or stability [13]. The NSs protein, the main interferon antagonist [14], carried only one conservative substitution, suggesting preserved function. NSm also retained conservative variation. Importantly, no amino acid changes were observed in the glycoproteins Gn and Gc at known neutralizing epitopes, suggesting that immune recognition and vaccine efficacy should remain unaffected [15].

## 4 Discussion

Although the outbreak genomes were highly conserved, the presence of a few adaptive polymerase changes suggests ongoing fine‐tuning of replication capacity under purifying selection. The persistence of lineage H in Senegal supports endemic maintenance rather than re‐emergence through importation. This pattern mirrors earlier findings from Mauritania (2020) and southern Africa, where lineage H was associated with increased replication efficiency and more severe clinical outcomes [6, 8].

During the 2020 Mauritania epidemic, which caused 78 confirmed human cases and 25 deaths, NSs gene sequences were identical to those of the 2020 Senegalese isolates from Fatick, yet the epidemiologic outcomes diverged sharply. While Mauritania experienced a severe epidemic with high fatality [16], Senegal reported only sporadic human cases without deaths [7]. This contrast highlights how viral genetics alone may not fully explain outbreak severity and underscores the potential influence of ecological, host‐related, and behavioral factors such as patterns of animal handling, slaughtering practices, and exposure to infected livestock, on Rift Valley fever dynamics [17].

Experimental studies have also shown that lineage H replicates more efficiently in vitro than other epidemic lineages (G and C) [8], supporting its apparent fitness advantage and capacity for rapid emergence under favorable ecological conditions. The combination of intrinsic viral fitness and extrinsic environmental factors, including vector density, livestock movement, and underlying population immunity levels, may therefore determine whether lineage H produces localized transmission or large‐scale epidemics. Although direct pre‐outbreak herd immunity data for the Saint‐Louis region in 2025 are missing, serological studies from northern Senegal offer meaningful context. In particular, Durand and colleagues reported an IgG seroprevalence of ~15.3% among resident small‐ruminant herds (*n* = 222) in the Saint‐Louis/Matam area, indicating a moderate level of natural herd immunity shaped by nomadic herd movements and seasonal vector availability [18]. This incomplete immunity may reduce, but not eliminate, viral amplification potential, thereby enabling the relatively “stable” but subtly evolving lineage H viruses (characterized by mostly conservative amino‐acid substitutions) to persist at low levels between outbreaks and re‐emerge when ecological, behavioral, or immunological conditions align.

The amino acids changes shared among strains circulating in Senegal in 2020, 2022, and 2025, indicating ongoing but limited evolutionary change within lineage H. Although functional assays were not conducted, the identified nonconservative substitutions occur within domains essential for polymerase activity, suggesting potential impacts on replication fidelity and viral fitness. These changes may fine‐tune polymerase conformation or replication efficiency while maintaining overall protein stability, as supported by the predominance of purifying selection across the genome. Importantly, no amino‐acid alterations were detected within the neutralizing epitopes of the Gn and Gc glycoproteins, indicating that antigenic recognition and vaccine efficacy are likely preserved.

The specific polymerase substitution identified in the 2025 strains warrants functional characterization to evaluate its impact on replication kinetics, polymerase processivity, and host–virus interactions. Such phenotypic studies—integrating in vitro and in vivo models—will be essential to determine whether these mutations subtly enhance replication efficiency or alter host immune modulation. Linking genomic data with phenotypic and immunological profiling will provide a more complete understanding of how small molecular changes translate into variations in pathogenicity and outbreak potential.

Clinically, the elevated case‐fatality rate observed during the 2025 outbreak aligns with prior lineage H epidemics, supporting a possible link between viral genetic background and disease severity [8]. This observation reinforces the need to integrate molecular findings with clinical and epidemiological data to better understand genotype–phenotype relationships in RVFV infections.

Lineage H has previously been associated with more severe outbreaks in Mauritania, South Africa, and Namibia. The 2025 epidemic in Senegal fits this pattern, with lineage H strains showing strong genetic continuity with earlier West African isolates and subtle amino‐acid changes in the L polymerase that may fine‐tune viral replication.

Together, these findings indicate that lineage H is now endemically established in West Africa, capable of causing recurrent outbreaks with varying severity. Genomic data reveal limited evolutionary change, with mostly conservative and a few nonconservative substitutions that spare the key Gn and Gc epitopes, suggesting that vaccine protection would not be compromised. Continued genomic surveillance—integrated with clinical, functional, and ecological monitoring—will be essential to detect further adaptive changes and to inform diagnostic and vaccine strategies aimed at mitigating future Rift Valley fever epidemics.

## Author Contributions

Moussa Moïse Diagne, Gamou Fall, Diawo Diallo, Abdourahmane Sow, and Oumar Faye conceived and coordinated the study. Moussa Moïse Diagne, Gamou Fall, and Ndeye Awa Ndiaye contributed to study design, data analysis, and manuscript writing. Gamou Fall, Abiboulaye Sall, Bocar Sow, Alioune Gaye, El Hadji Ndiaye, Mignane Ndiaye, Yoro Sall, Mamadou Aliou Barry, Ibou Gueye, Marie Henriette Dior Ndione, Boly Diop, Ousmane Cissé, Ibrahima Dia, Ousmane Faye, Mawlouth Diallo, Boubacar Diallo, Mamadou Ndiaye, Diawo Diallo, Abdourahmane Sow, and Oumar Faye supported outbreak investigation, diagnostics, and field coordination. Moussa Moïse Diagne, Ndeye Awa Ndiaye, Seynabou Mbaye Ba Souna Diop, Safiétou Sankhe, Mouhamed Kane, and Seynabou Ndiaye performed laboratory testing, sequencing, and genomic data curation. Ndeye Awa Ndiaye, Moussa Moïse Diagne, Aboubacry Gaye, Mamadou Sarr Ndao, and Cheikh Loucoubar contributed to data management and bioinformatics analysis. Moussa Moïse Diagne, Ndongo Dia, Boubacar Diallo, Abdourahmane Sow, Ibrahima Soce Fall, and Joseph R. A. Fitchett secured project funding and institutional support. Ibrahima Soce Fall, Abdourahmane Sow, Joseph R. A. Fitchett, Ndongo Dia, Mawlouth Diallo, and Oumar Faye provided strategic oversight, institutional coordination, and critical manuscript review.

## Conflicts of Interest

The authors declare no conflicts of interest.

## Supporting information


**Supporting Table S1:** Pairwise selection pressure analyses of RVFV S segment genomes.

**Supporting Table S2:** Pairwise selection pressure analyses of RVFV L segment genomes.

**Supporting Table S3:** Pairwise selection pressure analyses of RVFV M segment genomes.

## Data Availability

Consensus genomes (2025 Senegal) presented in the manuscript will be available shortly in GenBank. Raw FASTQ files and analysis scripts are available on request to Institut Pasteur de Dakar.
